# Supplementary material for: Chemogenetic inhibition of the medial prefrontal cortex reverses the effects of REM sleep loss on sucrose consumption
Source: eLife. 2016 Dec 6;5:e20269. doi: 10.7554/eLife.20269 (PMC5140266; doi:10.7554/eLife.20269)
Supplement: Supplementary file 1. — DOI: http://dx.doi.org/10.7554/eLife.20269.008 [file elife-20269-supp1.docx]

| **High fat diet** |  |  |
| --- | --- | --- |
|  | **Ingredients** | **grams** |
|  | lard | 330.0 |
|  | milk casein | 256.0 |
|  | α cornstarch | 160.0 |
|  | powdered cellulose | 66.1 |
|  | maltodextrin | 60.0 |
|  | sucrose | 55.0 |
|  | AN-93G-MX | 35.0 |
|  | soybean oil | 20.0 |
|  | AIN-93-VM | 10.0 |
|  | L-cystine | 3.6 |
|  | choline bitartrate | 2.5 |
|  | calcium carbonate | 1.8 |
|  |  | **1000.0 Total** |
| **White chocolate** |  |  |
|  | sucrose | 503.8 |
|  | lipids | 419.8 |
|  | protein | 75.6 |
|  | sodium | 0.8 |
|  |  | **1000.0 Total** |
